# Gambling problems in bipolar disorder in the UK: prevalence and distribution

**DOI:** 10.1192/bjp.bp.114.154286

**Published:** 2015-10

**Authors:** Lisa Jones, Alice Metcalf, Katherine Gordon-Smith, Liz Forty, Amy Perry, Joanne Lloyd, John R. Geddes, Guy M. Goodwin, Ian Jones, Nick Craddock, Robert D. Rogers

**Affiliations:** **Lisa Jones**, PhD, **Alice Metcalf**, Department of Psychiatry, University of Birmingham; **Katherine Gordon-Smith**, PhD, Department of Psychiatry, University of Birmingham, and National Centre for Mental Health, Medical Research Council (MRC) Centre for Neuropsychiatric Genetics and Genomics, Cardiff University; **Liz Forty**, PhD, National Centre for Mental Health, MRC Centre for Neuropsychiatric Genetics and Genomics, Cardiff University; **Amy Perry**, BSc, Department of Psychiatry, University of Birmingham; **Joanne Lloyd**, PhD, School of Psychology, Sport and Exercise Health, Staffordshire University, Stoke-on-Trent; **John R. Geddes**, MD, FRCPsych, **Guy M. Goodwin**, FMedSci, DPhil, FRCPsych, Department of Psychiatry, University of Oxford; **Ian Jones**, PhD, MRCPsych, **Nick Craddock**, PhD, FRCPsych, National Centre for Mental Health, MRC Centre for Neuropsychiatric Genetics and Genomics, Cardiff University; **Robert D. Rogers, PhD,** Department of Psychiatry, University of Oxford, and School of Psychology, Bangor University, UK

## Abstract

**Background**

North American studies show bipolar disorder is associated with elevated rates of problem gambling; however, little is known about rates in the different presentations of bipolar illness.

**Aims**

To determine the prevalence and distribution of problem gambling in people with bipolar disorder in the UK.

**Method**

The Problem Gambling Severity Index was used to measure gambling problems in 635 participants with bipolar disorder.

**Results**

Moderate to severe gambling problems were four times higher in people with bipolar disorder than in the general population, and were associated with type 2 disorder (OR = 1.74, *P* = 0.036), history of suicidal ideation or attempt (OR = 3.44, *P* = 0.02) and rapid cycling (OR = 2.63, *P* = 0.008).

**Conclusions**

Approximately 1 in 10 patients with bipolar disorder may be at moderate to severe risk of problem gambling, possibly associated with suicidal behaviour and a rapid cycling course. Elevated rates of gambling problems in type 2 disorder highlight the probable significance of modest but unstable mood disturbance in the development and maintenance of such problems.

With the expansion of commercial gambling throughout the UK, the opportunities and accessibility of gambling have also increased, reflecting similar trends in other jurisdictions.^1^ Problem gambling is excessive gambling behaviour that causes harm to the individual, their family and friends or to the wider community.^2^ The British Gambling Prevalence Survey (BGPS) of 2010 showed marginal increases in problem gambling within the UK between 2007 and 2010 (from 0.5% to 0.7%),^3^ but provided evidence that patterns of gambling participation across sectors of the community are changing – highlighting the need to understand better the individual differences or clinical factors that heighten the risk of gambling-related harm.^4^ North American studies have reported a particularly high prevalence of mood disorders, including bipolar disorder, among people who are problem gamblers,^5–8^ and an increased prevalence of problem gambling in individuals with bipolar disorder,^9^ which is associated with a poorer quality of life and prognosis.^10^ Mood disturbance in the form of hypomanic experiences is also associated with elevated rates of gambling problem symptoms,^11^ reflecting enhanced motivations to gamble for excitement and to regulate negative emotional states.^12^ Our study is the first to determine the prevalence of problem gambling in bipolar disorder in a UK sample, with a particular focus upon the severity of problem gambling risk reported in individuals with a diagnosis of type 2 bipolar disorder relative to type 1. The rich clinical data available on the sample allowed for an exploration of the associations between problem gambling and lifetime clinical variables in bipolar disorder.

## Method

Participants were drawn from the Bipolar Disorder Research Network (BDRN), a UK-wide ongoing research programme into the genetic and non-genetic determinants of affective disorders (www.bdrn.org). Its inclusion criteria are a main lifetime diagnosis of affective disorder, age 18 years or over, UK or Irish White ethnicity (owing to the focus on genetics) and ability to give written informed consent. Individuals are excluded from the BDRN if their mood disorder is a consequence of alcohol or substance misuse, medical illness, medication or an organic brain disorder, or if they are biologically related to another participant. Participants are recruited systematically through National Health Service (NHS) mental health services (community mental health teams and lithium clinics) and non-systematically using advertisements for volunteers on the BDRN website, leaflets, posters and media coverage about the research, and also through UK-based user-led charities such as Bipolar UK and Depression Alliance. Inclusion criteria for this study were a DSM-IV best-estimate lifetime diagnosis of bipolar disorder (types 1 and 2) or recurrent major depressive disorder (unipolar depression),^13^ and completion of the Problem Gambling Severity Index (PGSI).^2^ The research had NHS ethics approval (MREC/97/7/01) and research and development approval from all participating NHS trusts and health boards.

### Psychiatric assessment

Lifetime-ever clinical data for each individual in the BDRN study were collected by a trained BDRN interviewer (research psychologist or psychiatrist) using a semi-structured psychiatric interview, the Schedules for Clinical Assessment in Neuropsychiatry (SCAN).^14^ Further clinical data were gathered from participants' psychiatric case notes. Clinical interview and case-note data were combined to make best-estimate lifetime-ever diagnoses according to DSM-IV and ratings of lifetime-ever clinical characteristics. The Global Assessment Scale (GAS) was used to provide a measure of overall level of functioning during each participant's worst lifetime episodes of both depression and mood elevation;^15^ scores on this scale range from 1 (severe psychiatric disturbance) to 100 (good mental health). In cases of doubt, clinical ratings were made by at least two members of the research team unaware of each other's ratings, and consensus was reached through discussion where necessary. Interrater reliability was high. Mean kappa statistics were 0.85 for DSM-IV diagnoses and 0.81–0.99 for other key clinical categorical variables; mean intraclass correlation coefficients were 0.91–0.97 for key clinical continuous variables.

### Gambling assessment

Gambling behaviour was measured using the Problem Gambling Severity Index,^2^ a validated self-report instrument that measures gambling behaviour over the preceding 12 months. It is derived from the Canadian Problem Gambling Index and consists of nine items. For each item respondents answer on a four-point scale (0 never, 1 sometimes, 2 most of the time, 3 almost always). Total scores therefore range from 0 to 27, where 0 indicates no gambling problem, 1 or 2 a low risk of gambling problems, 3–7 moderate risk and 8 or over severe risk. The PGSI was mailed to 3500 BDRN participants in April 2011 and a reminder was sent 1 month later; 793 participants (23%) completed and returned the questionnaire.

### Statistical analysis

Following previous studies,^16^ we used two categories of problem gambling: moderate risk of gambling problems (PGSI score 3–7) and severe risk (PGSI score 8 or more). Statistical analyses were performed using SPSS version 21. To determine the clinical correlates of problem gambling in bipolar disorder, people rated as being at moderate or severe risk of problem gambling were compared with those at no or low risk (PGSI score <3) on a range of demographic and clinical variables using chi-squared tests, Mann–Whitney *U*-tests (owing to significant non-normal distributions of continuous variables) and multivariate binary logistic regression (using enter method). All tests were two-tailed and tested at a threshold of statistical significance of *P*<0.05.

## Results

A total of 750 participants met the inclusion criteria. The mean age at interview was 46.01 years (s.d. = 11.35) and 70.5% were women (*n* = 529). Over four-fifths (84.7%, *n* = 635) had bipolar disorder and 15.3% (*n* = 115) had major depressive disorder.

### Prevalence of problem gambling

Table 1 shows the prevalence of problem gambling in bipolar disorder and major depression. The prevalence of at least moderate risk of problem gambling in bipolar disorder was 10.6% (95% CI 8.21–12.99) and of severe risk was 2.7% (95% CI 1.44–3.96). In major depression the prevalence of at least moderate risk of problem gambling was 5.2% (95% CI 1.14–9.26) and of severe risk it was 0.9% (95% CI 0.83–2.63). The difference between bipolar disorder and major depression in the prevalence of both at least moderate risk and severe risk of problem gambling did not reach statistical significance (*P* = 0.087 and *P* = 0.336 respectively). The mean PGSI score in the moderate-risk group with bipolar disorder was 4.24 (95% CI 3.92–4.72; range 3–7, median 4) and in the severe-risk group with bipolar disorder it was 14.06 (95% CI 11.35–16.77; range 8–22, median 13). The sample size with severe risk of problem gambling with bipolar disorder was too small for further analysis (*n* = 17); therefore, all further analyses described below consider the combined group of participants with bipolar disorder and moderate to severe risk of gambling problems.

**Table 1:** Prevalence of problem gambling in participants with bipolar disorder and major depression

	Bipolar disorder group (*n* = 635) *n* (%)	Major depressive disorder (*n* = 115) *n* (%)	χ^2^	*P*
Risk of problem gambling				
Moderate or severe	67 (10.6)	6 (5.2)	3.153	0.087
Severe	17 (2.7)	1 (0.9)	1.358	0.336

### Gambling behaviours

Almost half of those with bipolar disorder at moderate or severe risk of problem gambling (*n* = 30; 46%) reported having gambled online in the preceding 12 months, and of these internet gamblers 57% (*n* = 17) reported that more than half of their gambling was conducted on the internet (25% of the total sample of those at moderate or severe risk; *n* = 17). Response frequencies for the individual PGSI items are shown in Table 2.

**Table 2:** Response frequencies of Problem Gambling Severity Index items in participants with bipolar disorder at moderate or severe risk of problem gambling (*n* = 67)

	*n* (%)^a^	95% CI of %
1. Have you ever bet on more than you could afford to lose?		
Never	21 (31)	27–35
At least sometimes	46 (69)	65–72
2. Have you ever needed to gamble with larger amounts to get the same feeling?		
Never	23 (34)	30–38
At least sometimes	44 (66)	61–69
3. Have you ever gone back to try to win back the money you had lost?		
Never	22 (33)	29–36
At least sometimes	45 (67)	63–71
4. Have you ever borrowed money or sold anything for money to gamble?		
Never	48 (72)	68–75
At least sometimes	19 (28)	24–32
5. Have you felt you might have a problem with gambling?		
Never	40 (60)	55–63
At least sometimes	27 (40)	36–44
6. Have people criticised your betting?		
Never	48 (72)	68–75
At least sometimes	19 (28)	24–32
7. Have you ever felt guilty about the way you gamble?		
Never	22 (33)	29–36
At least sometimes	45 (67)	63–71
8. Any health problems due to gambling?		
Never	29 (43)	39–47
At least sometimes	38 (57)	52–60
9. Any financial problems due to gambling?		
Never	34 (51)	46–54
At least sometimes	35 (49)	45–53

a. Numbers vary because of missing data.

### Demographic characteristics

Moderate or severe risk of problem gambling was significantly associated with several demographic characteristics (Table 3). The median age at interview was significantly younger in the moderate or severe risk group than in those at no or low risk (40 years *v.* 46 years) and moderate to severe risk of problem gambling was significantly associated with working in service industries (38% *v.* 27% in no or low-risk group) and being long-term unemployed (5% *v.* 1% in the no or low-risk group). However, there was no significant difference in gender distribution between the two risk groups (*P* = 0.09). Levels of education and marital history also did not significantly differ between the groups. There was no significant difference in the proportion of participants recruited systematically or non-systematically with and without moderate to severe risk of problem gambling.

**Table 3:** Demographic characteristics of participants with bipolar disorder categorised by severity of risk of problem gambling

	Risk of problem gambling			
	Moderate or severe (*n* = 67)^a^	No or low risk (*n* = 568)^a^	*U* or χ^2^	*P*
Age at interview, years				
Median	40	46	*U* = 15319.0	0.017*
IQR (range)	14 (18–66)	18 (18–76)		
Gender, *n* (%)				
Male	25 (37)	154 (27)	χ^2^ = 3.081	0.086
Female	42 (63)	414 (73)		
Marital history, *n* (%)				
Has married	52 (88)	436 (86)	χ^2^ = 0.174	0.841
Has never married	7 (12)	70 (14)		
Highest education, *n* (%)^b^				
No secondary education qualifications	5 (8)	44 (8)	χ^2^ = 2.371	0.499
CSE/O-level/GCSE	14 (21)	124 (22)		
A-level/AS-level	24 (36)	144 (25)		
Degree	22 (33)	206 (36)		
Highest occupation, *n* (%)				
Professional	37 (58)	374 (72)	χ^2^ = 7.696	0.021*
Service industry	24 (38)	139 (27)		
Never worked	3 (5)	7 (1)		
Method of recruitment, *n* (%)				
Systematic	16 (25)	132 (24)	χ^2^ = 0.007	1.000
Non-systematic	48 (75)	406 (76)		

IQR, interquartile range.

a. Numbers vary because of missing data.

b. Grades of UK secondary education are specified as GCSE, General Certificate of Secondary Education; O-level, ordinary level; A-level, advanced level; AS-level, advanced subsidiary level.

* P<0.05.

### Clinical characteristics

Moderate or severe risk of problem gambling was significantly associated with several lifetime clinical history variables (Table 4). Participants at moderate or severe risk were significantly more likely to have a DSM-IV diagnosis of type 2 bipolar disorder (40%) than those at no or low risk (28%). Of participants with type 2 disorder, 15% (*n* = 27) were rated as being at moderate or severe risk compared with 10% (*n* = 40) of those with type 1 disorder (OR = 1.74, 95% CI 1.03–2.92, *P* = 0.036). The mean PGSI score was 0.72 (95% CI 0.49–0.96; range 0–22, median 0) in the type 1 bipolar disorder group and 1.15 (95% CI 0.72–1.58; range 0–21, median 0) in the type 2 group – a statistically significant difference (*U* = 3794.5, *P* = 0.011).

**Table 4:** Lifetime-ever clinical features of participants with bipolar disorder categorised according to risk of problem gambling

	Risk of problem gambling			
	Moderate or severe (*n* = 67)^a^	No or low risk (*n* = 568)^a^	*U* or χ^2^	*P*
Clinical features, *n* (%)				
DSM-IV diagnosis				
Bipolar disorder type 1	40 (60)	409 (72)	χ^2^ = 4.382	0.036*
Bipolar disorder type 2	27 (40)	159 (28)		
Polarity of first affective episode				
Depression	48 (84)	328 (76)	χ^2^ = 1.945	0.184
(Hypo)mania	9 (16)	104 (24)		
History of rapid cycling	20 (56)	125 (33)	χ^2^ = 7.038	0.010*
History of psychotic features	33 (62)	298 (62)	χ^2^=0.000	1.000
History of suicidal ideation or attempt	59 (94)	433 (79)	χ^2^ = 7.604	0.004**
History of alcohol misuse	35 (61)	225 (47)	χ^2^ = 4.012	0.050*
History of smoking	43 (72)	263 (52)	χ^2^ = 8.029	0.006**
History of non-prescription drug misuse	21 (32)	138 (26)	χ^2^ = 1.262	0.297

Clinical features: median (IQR, range)				
Age at onset of illness, years	17 (7, 8–43)	21 (11, 5–68)	*U* = 12192.0	<0.001***
Number of episodes of (hypo)mania	10 (16, 1–100)	6 (9, 1–100)	*U* = 14348.5	0.044*
Number of episodes of depression	8 (15, 1–100)	8 (16, 0–100)	*U* = 14922.0	0.335
GAS score				
Worst episode of mood elevation	45 (20, 10–60)	33 (30, 9–65)	*U* = 13758.0	0.004**
Worst episode of depression	40 (15, 18–55)	40 (12, 3–71)	*U* = 15308.5	0.663

GAS, Global Assessment Scale; IQR, interquartile range.

a. Numbers vary because of missing data.

* *P*<0.05

** *P*<0.01

*** *P*<0.001.

The median age at onset of illness (defined as the age at first impairment due to affective illness) was significantly younger among participants at moderate or severe risk of problem gambling than among the no or low-risk group (17 years *v.* 21 years, *P*<0.001). Significantly more of those at moderate or severe risk had a history of rapid cycling, defined as four or more episodes of mania or hypomania in a 12-month period,^14^ than those not at risk (56% *v.* 33%, *P* = 0.010) and the median number of episodes of hypomania or mania was significantly higher (10 v. 6, *P* = 0.044). History of suicidal ideation or attempt was significantly more frequent among the moderate or severe risk group (94% *v.* 79% in the no or low-risk group, *P* = 0.004), as was history of alcohol misuse defined using DSM-IV criteria (61% *v.* 47%, *P* = 0.050) and regular smoking (72% *v.* 52%, *P* = 0.006). Finally, those at moderate or severe risk of problem gambling were significantly less impaired during their worst episode of mood elevation than those not at risk (GAS score 45 v. 33, *P* = 0.004). The levels of impairment during the worst episode of depression were similar in both risk groups.

Finally, binary logistic regression models showed that after controlling for age at interview and bipolar disorder type 1 or 2 diagnosis, the clinical history variables that significantly predicted the presence of moderate and severe risk of problem gambling over its absence were a history of rapid cycling (OR = 2.63, 95% CI 1.29–5.34, *P* = 0.008), a history of suicidal ideation or attempt (OR = 3.44, 95% CI 1.21–9.73, *P* = 0.02) and younger age at illness onset (OR = 0.94, 95% CI 0.90–0.98, *P* = 0.002).

## Discussion

In previous studies Lloyd *et al* found that people with a history of hypomanic experiences reported more gambling problems online,^11^ and that their gambling was driven by the desire to experience enjoyment and to regulate mood.^12^ The major finding presented here is that people with a diagnosis of type 2 bipolar disorder were at significantly higher risk of gambling problems than those with a diagnosis of type 1 disorder. The characteristic feature of type 2 bipolar disorder is the presence of hypomanic rather than manic symptoms and an absence of the psychotic symptoms often observed in type 1 disorder.^13,17^ Therefore, these data suggest that the characteristics of mild mood elevation involving enhanced reward focus, sleeplessness and distractibility constitute particular risk factors for problematic use of gambling services. In addition, our finding that a quarter of patients with gambling problems reported that more than half of their gambling in the past 12 months had involved the internet highlights the potential for gambling-related harm in people with bipolar disorder using internet gambling services that are available 24 h a day through fast-developing technologies.^18^

These observations sit within the broad picture of a relatively high prevalence of gambling problems in patients with bipolar disorder in the UK, with around 1 in 10 individuals with the disorder being at least at moderate risk of problem gambling. The BGPS is the third nationally representative survey to provide data on the 12-month prevalence of problem gambling in the UK.^3^ The BGPS 2010 reported a prevalence of 0.7% for severe risk of problem gambling in the general population and 2.5% for at least moderate risk.^3^ Consistent with nationwide surveys of the population of the USA,^8,18,19^ we found elevated rates of gambling problems in our UK sample of patients with bipolar disorder–specifically, that the 12-month prevalence of both severe and at least moderate risk of problem gambling is around four times higher in individuals with bipolar disorder than in the general population (3% and 11% respectively). These findings are also largely consistent with those from Canada: Kennedy *et al* reported a prevalence of 12% for at least moderate risk of problem gambling in individuals with bipolar disorder,^10^ and Quilty *et al* reported a prevalence of 3% for severe risk and 10% for moderate risk of problem gambling in bipolar disorder.^20^ Similarly, we found the prevalence of severe or moderate risk of problem gambling in participants with a diagnosis of major depressive disorder was elevated relative to figures for the general population (severe 0.9%, at least moderate 5.2%). However, this increase was not statistically significant, reflecting the relatively small sample size of patients with depression. Collectively, these data confirm the relatively strong associations between bipolar disorder and gambling problems, suggesting that the characteristic mood disturbance of bipolar disorder can have a powerful role in the development and maintenance of gambling problems.

### Demographic and clinical factors

More generally, our data suggest that patients with bipolar disorder who are at risk of problem gambling are likely to be younger and to have an earlier illness onset than patients at low risk, and also are more likely to work in service industries or be unemployed. In contrast to previous studies in the general population and in bipolar disorder which have shown a higher prevalence of problem gambling in men compared with women,^3,10^ no gender difference was observed. Therefore, gambling problems may be relatively common in women with bipolar disorder in the UK. Alcohol misuse in this bipolar disorder sample was significantly more prevalent among men than women (40% *v.* 29%, *P* = 0.02) as would be expected from UK general population prevalence figures,^21^ suggesting that the lack of expected gender difference is specific to gambling rather than a general predilection towards addiction among the women in our sample.

Even after controlling for bipolar type diagnoses, we found that rapid cycling and suicidal ideation or attempt were significantly associated with gambling problems. Rapid cycling was over 2.5 times more frequent in individuals at risk of problem gambling compared with individuals at low risk, and similarly those with gambling problems reported having had more lifetime episodes of hypomania or mania. The number of episodes of depression, however, was not significantly elevated. Those at moderate or severe risk of problem gambling were also 3.5 times more likely to have considered or attempted suicide. This is supported by Kennedy *et al*, who reported that people with gambling problems in a bipolar disorder sample in Canada were more than twice as likely to have been at higher suicide risk in the preceding month compared with those with no gambling problem.^10^ Suicide risk is known to be elevated in bipolar disorder,^17,22–24^ and our study demonstrates that comorbid gambling problems elevate this risk further. However, our data do not suggest that gambling problems are simply a marker of illness severity in bipolar disorder, as illustrated by significantly less functional impairment (i.e. higher GAS scores) in their worst episode of mood elevation among those at risk of problem gambling compared with those at low risk.

### Strengths and limitations

This study is the first to determine the prevalence of gambling problems in a UK sample with bipolar disorder, as well as exploring the associations between risk of problem gambling and lifetime clinical variables. Its strengths include the large, representative sample of patients with bipolar disorder and the rich clinical history data available concerning these patients. However, there were also several limitations. First, although it is widely used and validated, the PGSI is a self-report measure subject to a degree of social desirability and recall bias. Such bias was minimised at least to some extent because all questionnaires were completed in a private and confidential manner, encouraging honest reporting, and gambling behaviours were assessed over the previous 12 months only. Second, 23% of invited BDRN participants returned the PGSI questionnaire, which inevitably introduces responder bias to the data. It is difficult to know whether this bias over- or underestimates the prevalence of gambling problems. People who are currently gambling might be more likely to be interested in the research and complete the questionnaire; conversely, they might prefer not to disclose their gambling behaviours and thus not respond. However, the PGSI was included in a mail-shot with a number of other questionnaires, and responders completed all questionnaires, which reduces the likelihood that the decision to respond was particularly influenced by the inclusion of the PGSI. Third, the study was limited by the size of the sample at severe risk of problem gambling in the bipolar disorder group (*n* = 17), which was insufficient for further analysis. The sample size for individuals with major depression was also small (*n* = 115), so we can have less confidence in the estimated prevalence rates of gambling problems in this group. Fourth, given the exploratory nature of the study we did not control for multiple statistical tests across variables. Therefore, our findings require independent replication. However, some of our statistically significant findings would stand up to correction for multiple comparisons; for example, the associations of moderate and severe risk of problem gambling with suicidal ideation or attempts and younger age at illness onset. Finally, the cross-sectional design of the study does not allow us to make inferences about causality, that is, whether mood dysregulation in bipolar disorder contributes to problem gambling, or whether problem gambling is used as a way of regulating mood as suggested by Lloyd *et al.*^12^

### Future research

Understanding the temporal relationship between bipolar disorder and problem gambling, and the mechanisms underlying the links between these disorders, requires longitudinal studies. For example, in our study the association between lifetime rapid cycling and gambling problems in the preceding 12 months can be explained by the presence of hypomanic or manic episodes during this period; however, the cross-sectional design makes this hard to assess. Future research would also benefit from assessing motivations for gambling in bipolar disorder. These findings require replication in large, independent samples of people with bipolar disorder. All participants in this study were of UK White ethnicity, and thus future studies should explore problem gambling in other ethnic groups with bipolar disorder.

### Implications for clinical practice

Problem gambling, unlike alcohol and drug misuse, is currently not screened for when assessing patients with bipolar disorder as part of routine clinical practice in the UK. Findings from this study can be used to inform clinicians not only of the increased risk of problem gambling in bipolar disorder, but also of its association with type 2 disorder, suicidal behaviour and an unstable rapid cycling illness course. Clinicians should consider routinely assessing gambling problems in patients with bipolar disorder.
